# Cisplatin-Induced Nephrotoxicity Attenuation by *Schizophyllum commune* Through Regulating Mitochondria-Associated Signaling, Apoptosis, Autophagy, and PINK1/Parkin-Mediated Mitophagy

**DOI:** 10.3390/ijms27125302

**Published:** 2026-06-11

**Authors:** Yu-Wen Sun, Te-Kai Sun, Wen-Ping Jiang, Guan-Jhong Huang

**Affiliations:** 1 Department of Chinese Pharmaceutical Sciences and Chinese Medicine Resources, College of Chinese Medicine, China Medical University, Taichung 404333, Taiwan; 2 School of Pharmacy, China Medical University, Taichung 404333, Taiwanwpjiang@cmu.edu.tw (W.-P.J.); 3 Department of Food Nutrition and Healthy Biotechnology, Asia University, Taichung 41354, Taiwan

**Keywords:** *Schizophyllum commune*, acute kidney injury, cisplatin, oxidative stress, mitochondria, mitophagy

## Abstract

Associated with high morbidity and mortality, cisplatin-induced acute kidney injury (AKI) is a common clinical complication characterized by oxidative stress, inflammation, and mitochondria-associated signaling. Although multiple signaling pathways have been implicated in AKI progression, effective interventions targeting these complex mechanisms are still lacking. As a medicinal fungus with antioxidant and anti-inflammatory properties, *Schizophyllum commune* (SC) has shown potential biological activities; however, its renoprotective effects in cisplatin-induced AKI remain unclear. Therefore, this study aimed to investigate SC’s protective effects and underlying mechanisms in a cisplatin-induced AKI mouse model. SC treatment improved renal function and attenuated histopathological damage. It reduced oxidative stress and inflammatory responses, as evidenced by the modulation of malondialdehyde (MDA), glutathione (GSH), nitric oxide (NO), and pro-inflammatory cytokines. Mechanistically, SC regulated multiple signaling pathways, including mitogen-activated protein kinase (MAPK), toll-like receptor 4/nuclear factor kappa B (TLR4/ NF-κB), PI3K/AKT, nuclear factor erythroid 2–related factor 2/heme oxygenase-1 (Nrf2/HO-1), and the calcium/calmodulin-dependent protein kinase kinase–AMP-activated protein kinase–sirtuin 1 (CaMKK–AMPK–Sirt1) axis. In addition, SC modulated apoptosis, autophagy, and PTEN-induced kinase 1 (PINK1)/Parkin-mediated mitophagy, suggesting improved mitochondrial homeostasis. These findings indicate that SC exerts renoprotective effects and may contribute to cisplatin-induced nephrotoxicity mitigation strategies.

## 1. Introduction

Defined by a rapid decline in renal function over a short period, acute kidney injury (AKI) is a common clinical syndrome with significant mortality risk. Based on etiology, AKI can be classified into three major categories: prerenal (reduced renal perfusion), intrinsic (renal parenchymal injury), and postrenal (urinary tract obstruction). Common causes include severe dehydration, shock, and sepsis, the use of nephrotoxic drugs or contrast agents, and obstruction resulting from urinary calculi or benign prostatic hyperplasia. The pathogenesis of AKI is highly complex and involves multiple interacting pathological processes, including oxidative stress, inflammation, mitochondria-associated signaling, apoptosis, and autophagy [1,2,3]. Epidemiological studies indicate that approximately 13.3 million AKI cases occur globally each year, with about 85% of these occurring in developing countries. Although a direct causal relationship between AKI and mortality has not been fully established, AKI is strongly associated with an increased risk of death, accounting for an estimated 1.7 million deaths annually [4]. Furthermore, AKI is associated with various long-term adverse renal outcomes, including an increased risk of AKI recurrence, progression to chronic kidney disease (CKD), and acceleration to end-stage renal disease (ESRD), thereby leading to reduced quality of life, functional impairment, and substantial long-term healthcare costs [5]. In addition to renal outcomes, AKI also impacts overall health status. Studies have demonstrated that AKI reduces health-related quality of life and may result in new disabilities in previously healthy children following AKI-associated infection, as well as long-term neurocognitive and behavioral impairments [6]. Moreover, AKI is associated with an increased risk of dementia in adults, with this risk rising significantly with age [7].

Due to the kidney’s critical role in drug metabolism and excretion, it is particularly susceptible to drug- and chemical-induced toxicity [8], and nephrotoxicity remains a major limitation in the clinical use of certain antibiotics, anticancer chemotherapeutic agents, and diagnostic reagents [9]. Common nephrotoxic agents in clinical practice include antimicrobial drugs, chemotherapeutic agents, calcineurin inhibitors, and contrast media [10]. Among the commonly used nephrotoxic chemotherapeutic agents, cisplatin, a platinum-based anticancer drug with broad-spectrum antitumor activity, has been widely used to treat various solid malignancies, including cancers of the lung, breast, bladder, ovary, testis, and head and neck [11]. Cisplatin induces renal tubular cell injury through multiple molecular mechanisms. Its anticancer activity is primarily mediated by binding to DNA and inducing DNA damage, thereby inhibiting both nuclear and mitochondrial DNA replication and activating multiple cellular signaling pathways, ultimately leading to cell necrosis or apoptosis [12,13,14].

*Schizophyllum commune* (SC) is an edible medicinal fungus known for its diverse bioactive properties, particularly its immunomodulatory, anti-inflammatory, and therapeutic effects. Its major bioactive components, especially polysaccharides, exhibit significant immunomodulatory, anti-inflammatory, antioxidant, and pharmacological activities [15]. SC has been shown to enhance immune responses by promoting T cell, B cell, and macrophage activation, as well as stimulating cytokine production while concurrently suppressing pro-inflammatory mediators such as tumor necrosis factor-α (TNF-α), interleukin (IL)-6, and IL-1β. Furthermore, bioactive polysaccharides isolated from SC have been shown to interact with signaling pathways such as mitogen-activated protein kinase (MAPK), nuclear factor kappa B (NF-κB), and phosphoinositide 3-kinase/protein kinase B (PI3K/AKT), which are associated with suppressing tumor progression, regulating apoptotic activity, and maintaining gut microbial homeostasis [16]. In addition, SC exhibits a broad range of biological activities, including antioxidant, antiviral [17], hypoglycemic, and angiotensin-converting enzyme inhibitory effects [18]. It may alleviate oxidative stress by reducing reactive oxygen species (ROS) accumulation and improving metabolic function through nuclear factor erythroid 2-related factor 2/heme oxygenase-1 (Nrf2/HO-1) pathway activation [19]. Beyond its therapeutic potential, SC also exhibits anti-photoaging, anti-tyrosinase, and skin-protective properties, as well as the ability to enhance enzyme stability and facilitate transdermal drug delivery, highlighting its promise as a functional food and therapeutic agent [20,21].

Nitric oxide (NO) is not only an inflammatory mediator but also a regulator of programmed cell death. Depending on its concentration and cellular context, NO-induced cell death may manifest as apoptosis or necrosis, with predominantly pro-apoptotic effects in renal tissues [22]. Mitochondria-associated signaling, including mitochondrial permeability transition pore opening, reactive oxygen and nitrogen species (RONS) generation, and cytochrome c release, is considered to be a major mechanism underlying NO-mediated apoptosis [23]. The PTEN-induced kinase 1 (PINK1)/Parkin-mediated mitophagy pathway is considered to be a major mechanism of mitophagy under cellular stress. Under normal conditions, PINK1 is imported into the mitochondrial inner membrane in a membrane potential-dependent manner and is subsequently rapidly degraded by the inner membrane protease PARL; however, when mitochondria undergo depolarization due to oxidative stress or toxic stimuli, PINK1 fails to be translocated into the inner membrane and instead accumulates on the outer mitochondrial membrane, where it becomes activated. Activated PINK1 subsequently phosphorylates ubiquitin and recruits the E3 ubiquitin ligase Parkin to the mitochondrial surface, leading to its activation and the polyubiquitination of outer mitochondrial membrane proteins. These ubiquitin-modified proteins are then recognized by the autophagy adaptor p62 (SQSTM1), which interacts with LC3 through its LC3-interacting region, thereby facilitating the sequestration of damaged mitochondria into autophagosomes and their subsequent delivery to autolysosomes for degradation, completing the mitophagy process [24,25]. PINK1/Parkin-mediated mitophagy alleviates renal injury by removing damaged mitochondria and suppressing mitochondrial ROS accumulation, NLRP3 inflammasome activation, and apoptosis [26]. However, excessive mitophagy may also be detrimental, as mitochondrial depletion can result in reduced ATP production, energy deficiency, and increased cell death [27]. Given the diverse biological activities and functional food potential of SC, it may be a promising adjunctive strategy for alleviating AKI. Accordingly, this study was conducted to evaluate the renoprotective effects of SC in a cisplatin-induced nephrotoxicity mouse model and to investigate the underlying mechanisms associated with renal injury.

## 2. Results

### 2.1. SC Attenuates Renal Injury and Improves Renal Function in Cisplatin-Treated Mice

**Figure 1:**
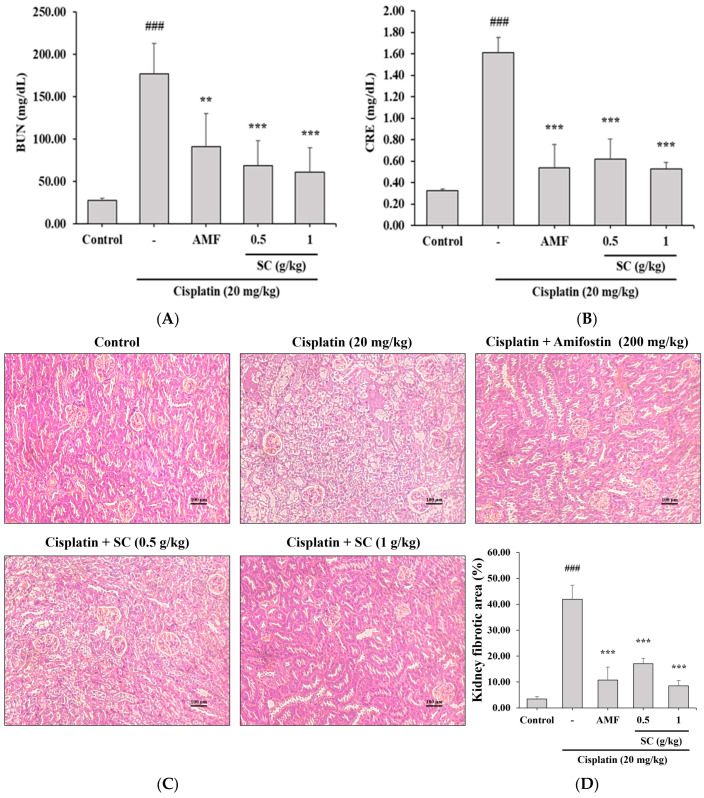
SC attenuates renal injury and improves renal function in cisplatin-treated mice. Mice were orally administered SC once daily for 10 consecutive days. On day 7, AKI was induced by a single intraperitoneal injection of cisplatin (20 mg/kg) 1 h after SC administration, and mice were sacrificed on day 10. BUN (**A**) and CRE (**B**) levels were measured. Kidney tissues were subjected to H&E staining for histopathological evaluation (**C**), and tubular injury scores were assessed (**D**). Representative histological sections (200× magnification) are shown, with tubular cell necrosis indicated by arrows. Data are presented as mean ± SD (*n* = 6). ^###^ *p* < 0.001 vs. control group; ** *p* < 0.01 and *** *p* < 0.001 vs. cisplatin group. The bars represent 100 μm.

Blood urea nitrogen (BUN) and creatinine (CRE) are key biomarkers for evaluating renal function. As shown in Figure 1A,B, cisplatin administration significantly increased serum CRE and BUN levels, indicating impaired renal function. In contrast, treatment with SC (0.5 and 1 g/kg) markedly attenuated CRE and BUN elevations, suggesting a protective effect against cisplatin-induced AKI. As a clinically used cytoprotective agent, amifostine (AMF) was included as a positive control and similarly reduced CRE and BUN levels. AMF is a cytoprotective agent that is widely used to reduce cisplatin-induced toxicity, including nephrotoxicity [28,29]. Histopathological examination was further performed to evaluate structural changes in the kidneys (Figure 1C). No obvious histopathological abnormalities were observed in the control group, and renal tubules and glomeruli remained structurally intact. In contrast, the cisplatin-treated group showed severe pathological alterations, including tubular epithelial cell injury, brush border loss, cellular swelling, and vacuolar degeneration, accompanied by tubular dilation, necrosis, and inflammatory cell infiltration. Notably, these histopathological abnormalities were markedly alleviated in the SC-treated groups, with improved tubular integrity and reduced necrosis and inflammation. Tubular injury scores (Figure 1D) were consistently significantly lower in SC-pretreated mice than in the cisplatin group. Collectively, these results demonstrate that SC effectively ameliorates cisplatin-induced renal dysfunction and structural damage, indicating its protective role against AKI.

### 2.2. Effects of SC on Body Weight and Kidney Index in Cisplatin-Treated Mice

The kidney-to-body weight ratio serves as an indicator of renal injury severity in cisplatin-treated mice. As indicated in Table 1, compared to the control group, cisplatin-treated mice exhibited a significant reduction in body weight, accompanied by a notable increase in kidney index. In contrast, SC-treated mice showed marked resistance to cisplatin-induced nephrotoxicity, as evidenced by a significant reduction in the kidney index.

### 2.3. SC Alleviates Cisplatin-Induced Elevation of Serum NO and Pro-Inflammatory Cytokines

**Figure 2:**
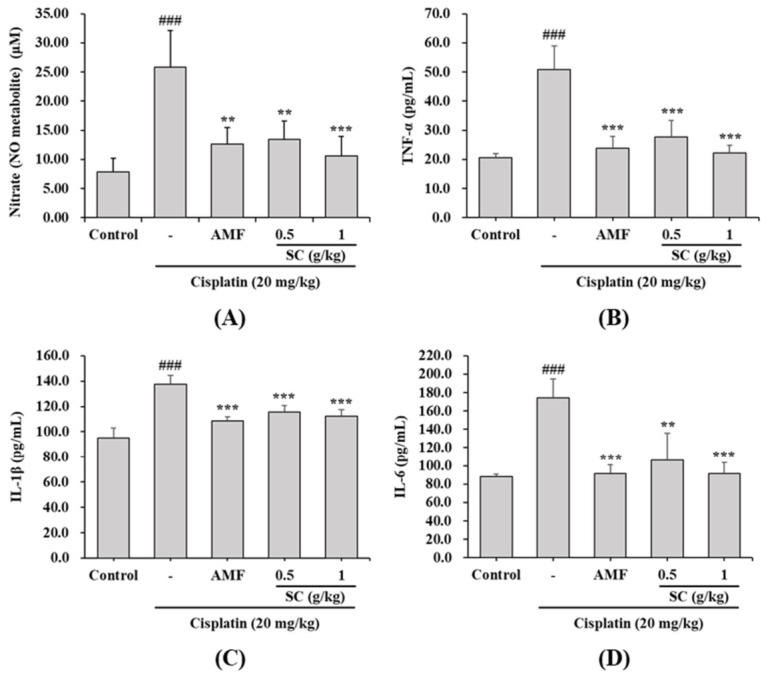
SC alleviates NO and pro-inflammatory cytokine levels in cisplatin-induced nephrotoxicity. NO (**A**), TNF-α (**B**), IL-1β (**C**), and IL-6 (**D**) serum was quantified. Nitrite concentration was determined using the Griess reaction, while ELISA measured cytokine levels. Data are expressed as mean ± SD (*n* = 6). ^###^ *p* < 0.001 vs. control group; ** *p* < 0.01 and *** *p* < 0.001 vs. cisplatin group.

NO and pro-inflammatory cytokines, including TNF-α, IL-1β, and IL-6, are key mediators involved in inflammatory responses; they are widely used as renal inflammation indicators. Cisplatin administration markedly elevated serum levels of NO metabolite, TNF-α, IL-1β, and IL-6 relative to those observed in the control group, indicating a pronounced inflammatory response (Figure 2A–D). Notably, treatment with SC markedly attenuated these elevations. Similarly, AMF also significantly reduced the NO and pro-inflammatory cytokine levels. Specifically, SC significantly reduced serum NO levels, which reflect nitrosative stress, as well as TNF-α, IL-1β, and IL-6, which are central regulators of inflammatory signaling. These observations suggest that SC might reduce the inflammatory responses induced by cisplatin in vivo.

### 2.4. Attenuation of Oxidative Stress in Cisplatin-Induced Nephrotoxicity

**Figure 3:**
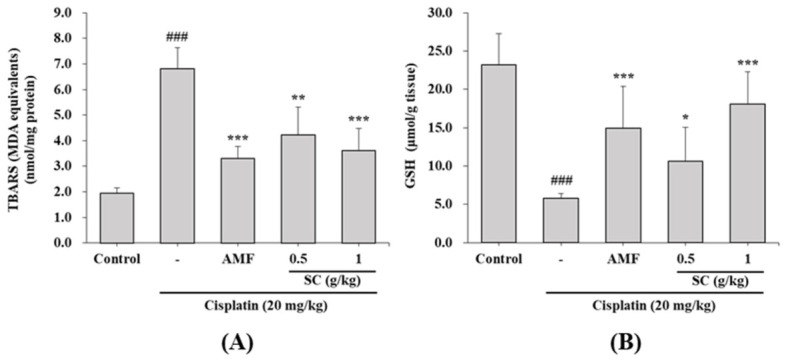
Attenuation of oxidative stress in cisplatin-induced nephrotoxicity. Levels of TBARS (MDA equivalents) (**A**) and GSH (**B**) in kidney tissues were determined. TBARS levels were measured as an index of lipid peroxidation, while GSH levels were assessed using the DTNB assay. Data are expressed as mean ± SD (*n* = 6). ^###^ *p* < 0.001 vs. control group; * *p* < 0.05, ** *p* < 0.01 and *** *p* < 0.001 vs. cisplatin group.

Oxidative stress is a key mechanism involved in cisplatin-induced renal injury. To determine whether SC treatment modulates oxidative stress in this model, levels of thiobarbituric acid reactive substances (TBARS)—expressed as Malondialdehyde (MDA) equivalents—and Glutathione (GSH) in kidney tissues were evaluated. As shown in Figure 3A,B, cisplatin administration significantly increased TBARS levels and markedly decreased GSH levels compared with the control group, indicating enhanced oxidative stress. Notably, both SC and AMF treatments effectively attenuated these alterations, as evidenced by a significant reduction in TBARS levels and GSH content restoration. These findings suggest that SC alleviates cisplatin-induced oxidative stress in kidney tissues.

### 2.5. MAPK and TLR4/NF-κB Signaling Pathway Inhibition in Cisplatin-Induced Nephrotoxicity

**Figure 4:**
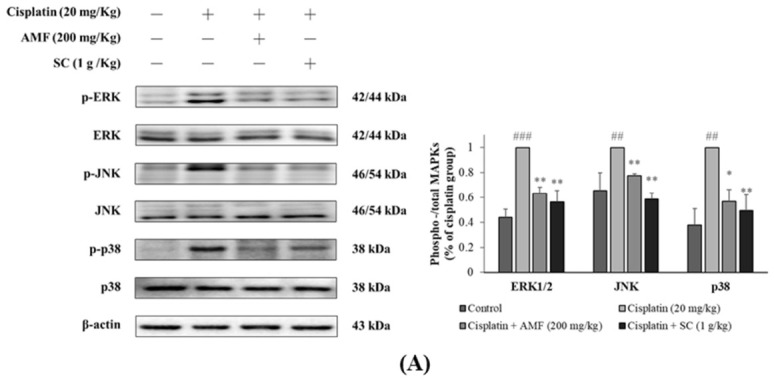

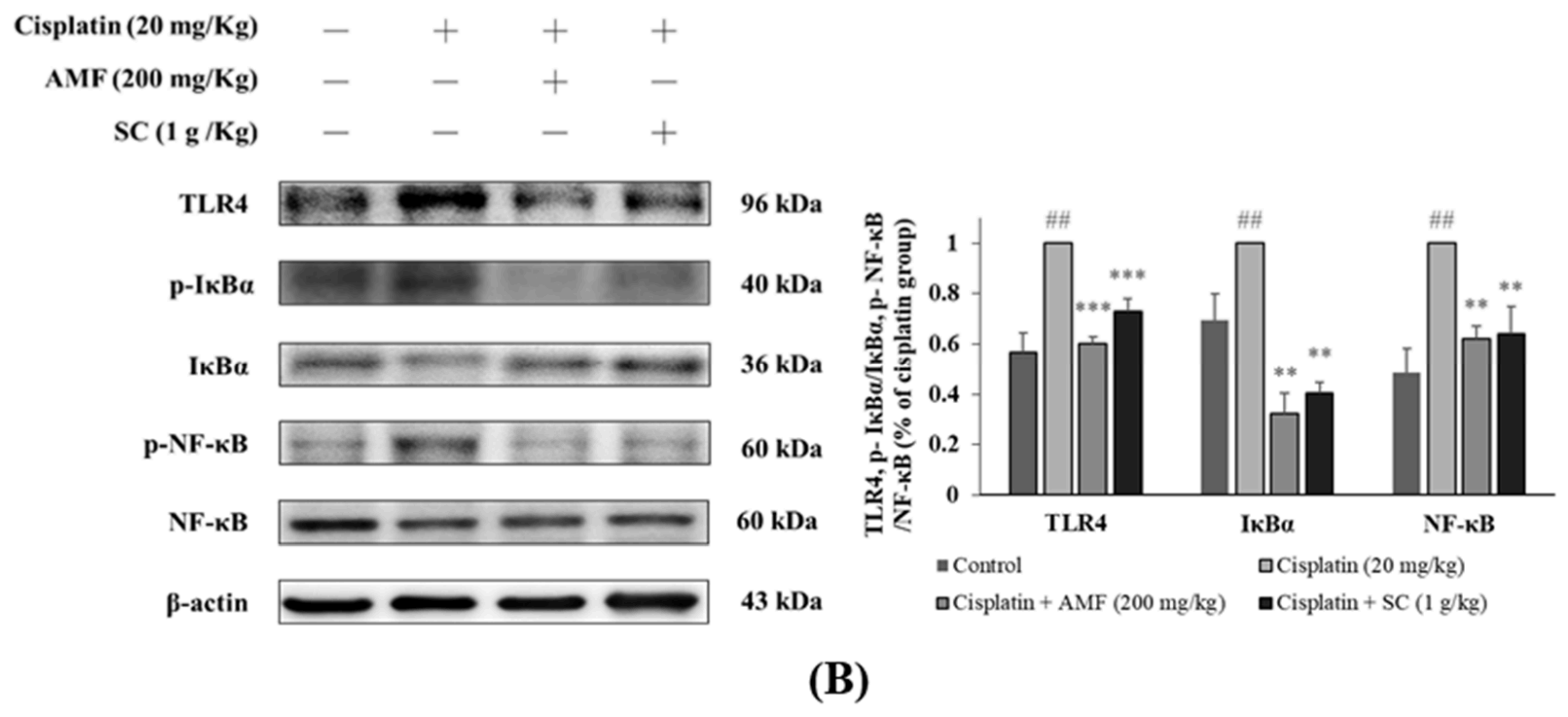
SC inhibits MAPK and TLR4/NF-κB signaling pathways in cisplatin-induced nephrotoxicity. Protein expression levels of MAPK (**A**) and TLR4, IκBα, and NF-κB (**B**) in renal tissues were determined by Western blot following cisplatin treatment. Protein bands were quantified by densitometric analysis. Data are presented as mean ± SD. The experiment was repeated three times (*n* = 3). ^##^ *p* < 0.01 and ^###^ *p* < 0.001 vs. control group; * *p* < 0.05, ** *p* < 0.01 and *** *p* < 0.001 vs. cisplatin group. Symbols “−” and “+” indicate the absence or presence of SC.

The MAPK and TLR4/NF-κB signaling pathways play critical roles in mediating inflammatory responses during cisplatin-induced renal injury. Phosphorylation of MAPK pathway proteins, including ERK1/2, JNK, and p38, was significantly elevated following cisplatin administration relative to untreated controls, indicating MAPK signaling activation (Figure 4A). In contrast, both SC and AMF treatments markedly suppressed the phosphorylation of ERK1/2, JNK, and p38, suggesting MAPK pathway activation inhibition. As shown in Figure 4B, cisplatin administration significantly upregulated TLR4, NF-κB, and IκBα expression, indicating TLR4/NF-κB signaling pathway activation. Notably, treatment with SC and AMF effectively attenuated these increases, as evidenced by reduced TLR4, NF-κB, and IκBα expression levels. These results suggest that SC alleviates cisplatin-induced inflammatory responses by suppressing MAPK and TLR4/NF-κB signaling pathway activation.

### 2.6. Renal Antioxidant Defense Restoration and Nrf2/HO-1 Signaling Pathway Regulation in Cisplatin-Induced Nephrotoxicity

**Figure 5:**
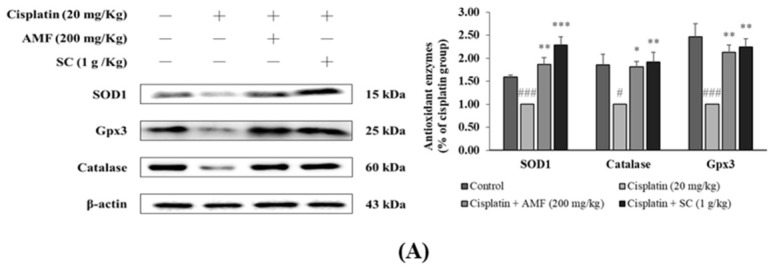

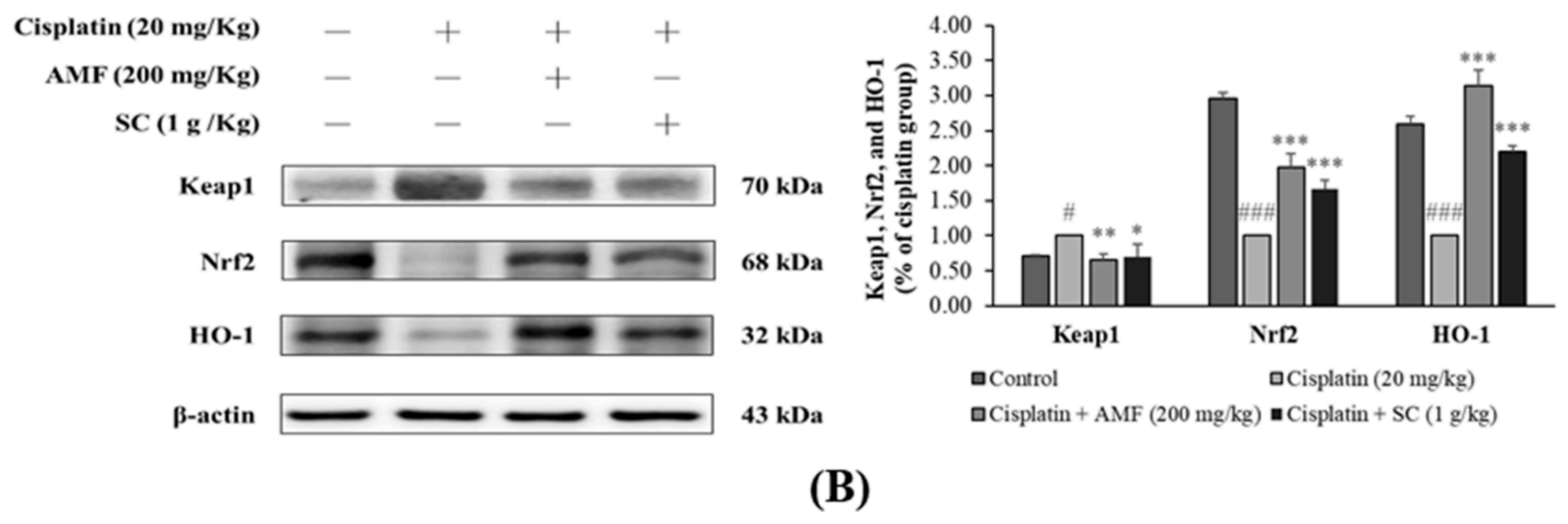
Restoration of antioxidant defense and Nrf2/HO-1 signaling in cisplatin-induced nephrotoxicity. Protein expression levels of antioxidant enzymes, including catalase, SOD1, and GPx3 (**A**), and Keap1, Nrf2, and HO-1 (**B**), in renal tissues were analyzed by Western blot following cisplatin treatment. Protein bands were quantified by densitometric analysis. Data are presented as mean ± SD. The experiment was repeated three times (*n* = 3). ^#^ *p* < 0.05 and ^###^ *p* < 0.001 vs. control group; * *p* < 0.05, ** *p* < 0.01 and *** *p* < 0.001 vs. cisplatin group. Symbols “−” and “+” indicate the absence or presence of SC.

As a major antioxidant defense regulator, Nrf2 is critically involved in oxidative stress responses and cellular redox equilibrium. Catalase, SOD1, and GPx3 protein expression levels were markedly decreased following cisplatin treatment, indicating suppression of the renal antioxidant defense system relative to untreated controls (Figure 5A). In contrast, SC and AMF treatment markedly reversed the expression of these antioxidant enzymes, indicating improved antioxidant capacity. As shown in Figure 5B, cisplatin treatment disrupted the Nrf2/HO-1 signaling pathway, as indicated by altered Keap1, Nrf2, and HO-1 expression. Notably, SC and AMF treatments effectively reversed these changes, as evidenced by the increased expression of Nrf2 and its downstream target, HO-1, and modulation of Keap1 expression. These findings indicate that SC enhances antioxidant defense and alleviates cisplatin-induced oxidative stress by regulating the Nrf2/HO-1 signaling pathway.

### 2.7. CaMKK–AMPK–Sirt1 Signaling Axis Modulation in Cisplatin-Induced Nephrotoxicity

**Figure 6:**
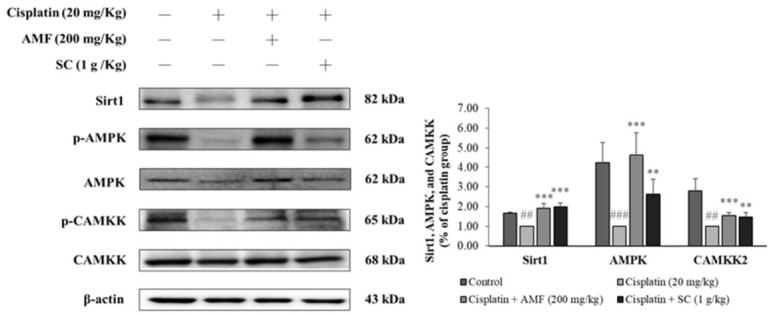
Modulation of the CaMKK–AMPK–Sirt1 signaling axis in cisplatin-induced nephrotoxicity. Sirt1, p-CaMKK, and p-AMPK protein expression levels in renal tissues were analyzed by Western blot following cisplatin treatment. Protein bands were quantified by densitometric analysis. Data are presented as mean ± SD. The experiment was repeated three times (*n* = 3). ^##^ *p* < 0.01 and ^###^ *p* < 0.001 vs. control group; ** *p* < 0.01 and *** *p* < 0.001 vs. cisplatin group. Symbols “−” and “+” indicate the absence or presence of SC.

AMPK is a key energy sensor that regulates cellular energy homeostasis by modulating NAD^+^ metabolism and sirtuin 1 (Sirt1) activity; it can be activated by upstream CaMKK in a Ca^2+^-dependent manner [30,31]. Figure 6 illustrates how cisplatin administration markedly reduced renal protein expression of p-CaMKK, p-AMPK, and Sirt1 relative to the control group, reflecting CaMKK–AMPK–Sirt1 signaling axis suppression. In contrast, treatment with SC and AMF significantly restored p-CaMKK, p-AMPK, and Sirt1 expression. These findings indicate that SC modulates the CaMKK–AMPK–Sirt1 signaling pathway in cisplatin-induced nephrotoxicity.

### 2.8. PI3K/AKT Signaling Pathway Regulation in Cisplatin-Induced Nephrotoxicity

**Figure 7:**
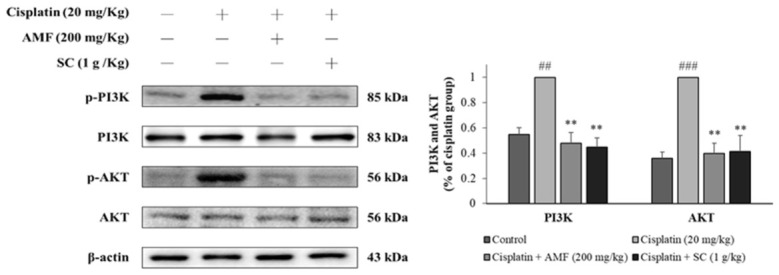
Regulation of the PI3K/AKT signaling pathway in cisplatin-induced nephrotoxicity. p-PI3K and p-AKT protein expression levels in renal tissues were analyzed by Western blot following cisplatin treatment. Protein bands were quantified by densitometric analysis. Data are presented as mean ± SD. The experiment was repeated three times (*n* = 3). ^##^ *p* < 0.01 and ^###^ *p* < 0.001 vs. control group; ** *p* < 0.01 vs. cisplatin group. Symbols “−” and “+” indicate the absence or presence of SC.

PI3K/AKT signaling has been implicated in regulating cellular stress adaptation, inflammatory responses, and apoptosis under pathological conditions [32,33]. In the present study, cisplatin administration significantly increased PI3K and AKT phosphorylation levels in renal tissues (Figure 7). This finding is consistent with previous reports showing elevated PI3K/AKT phosphorylation following cisplatin exposure during acute renal injury [34]. Moreover, it has been demonstrated that PI3K/AKT phosphorylation suppression attenuates cisplatin-induced acute kidney injury [35]. Accordingly, in our model, treatment with SC and AMF markedly reduced PI3K and AKT phosphorylation levels.

### 2.9. Apoptosis, Autophagy, and PINK1/Parkin-Mediated Mitophagy Regulation in Cisplatin-Induced Nephrotoxicity

**Figure 8:**
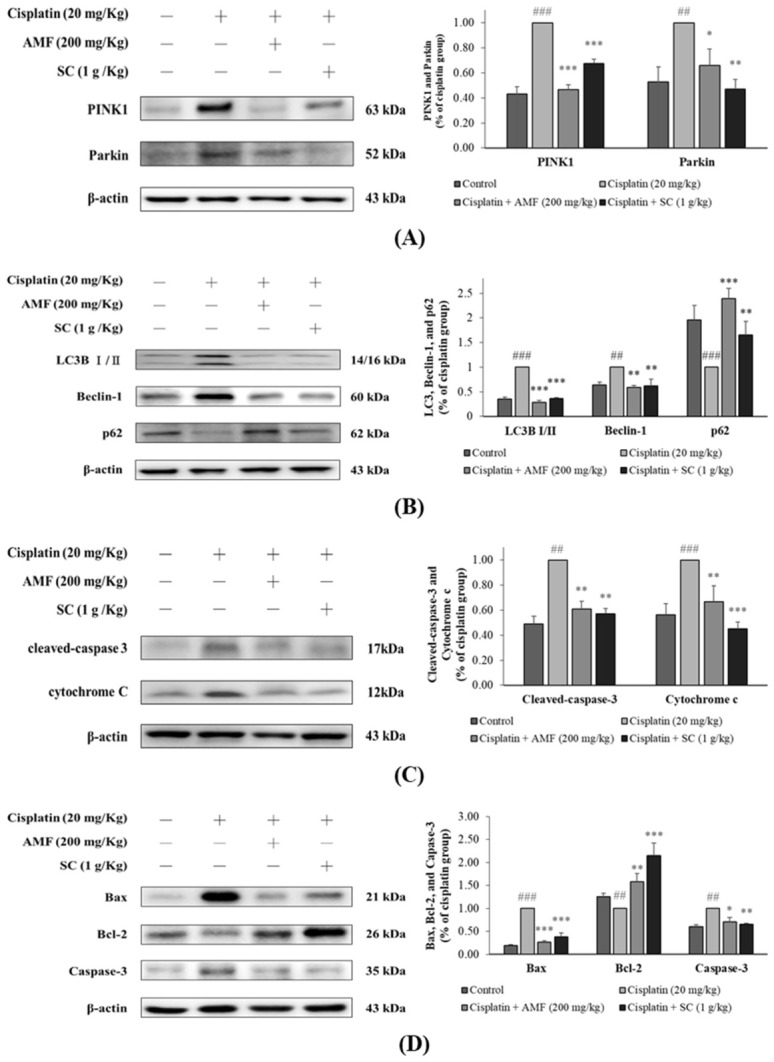

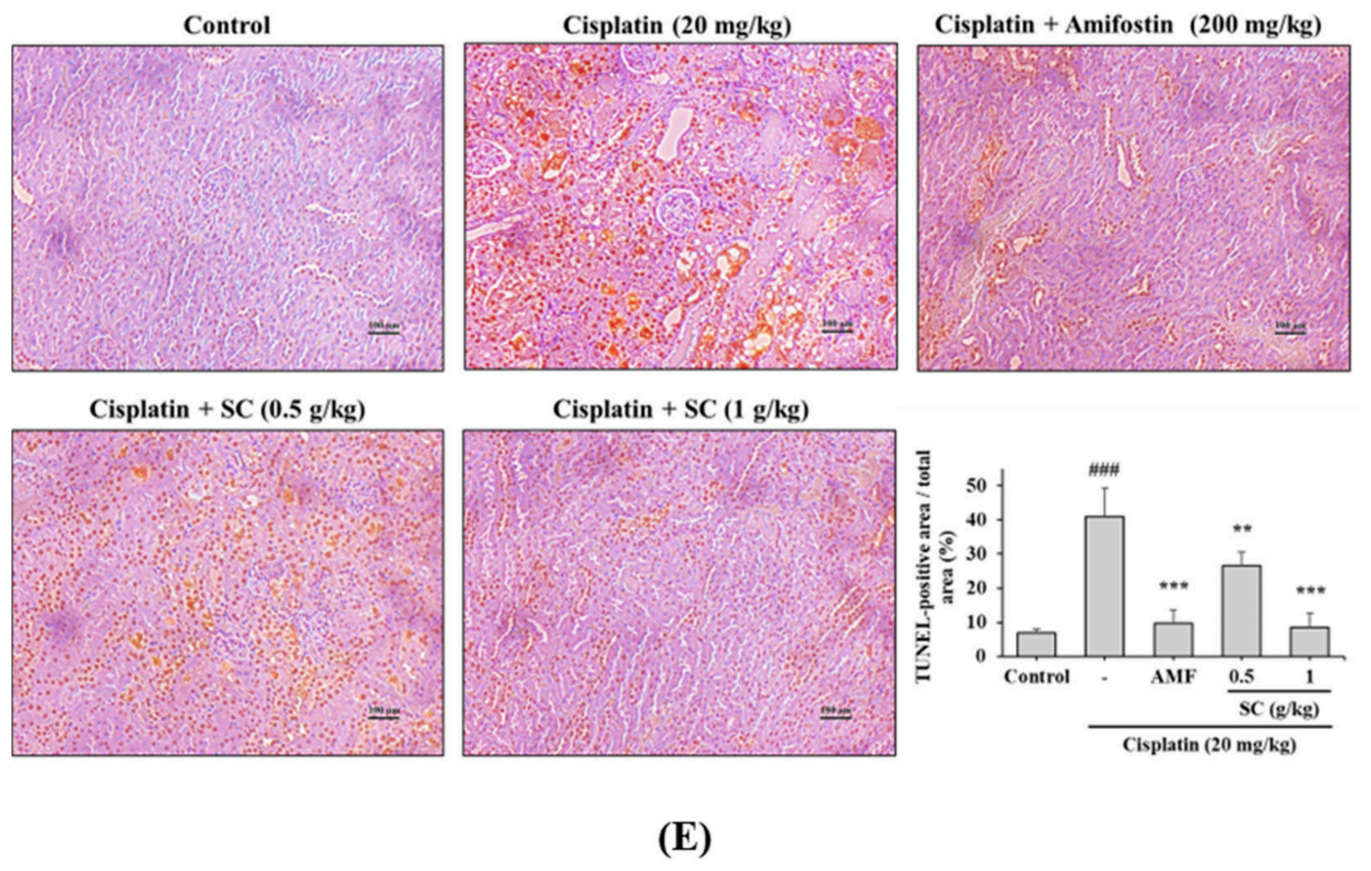
Regulation of apoptosis, autophagy, and PINK1/Parkin-mediated mitophagy in cisplatin-induced nephrotoxicity. In renal tissues, the expression levels of PINK1 and Parkin (**A**), autophagy-related proteins LC3, Beclin-1, and p62 (**B**), cytochrome c and cleaved caspase-3 (**C**), and apoptosis-related proteins Bax, Bcl-2, and caspase-3 (**D**) were analyzed by Western blot following cisplatin treatment. TUNEL staining (200×) was performed to evaluate apoptotic cell death in renal tissues (**E**). TUNEL-positive area was quantified using an image analyzer and expressed as TUNEL-positive area/total area (%). Data are presented as mean ± SD (*n* = 5 for E). The experiment was repeated three times for (**A**–**D**) (*n* = 3). ^##^ *p* < 0.01 and ^###^ *p* < 0.001 vs. control group; * *p* < 0.05, ** *p* < 0.01 and *** *p* < 0.001 vs. cisplatin group. The bars represent 100 μm. Symbols “−” and “+” indicate the absence or presence of SC.

Mitophagy is a selective form of autophagy responsible for removing dysfunctional or damaged mitochondria [36]. Mitophagy, autophagy, and apoptosis are critical processes involved in cisplatin-induced renal injury. As shown in Figure 8A, cisplatin administration significantly increased the protein expression levels of PINK1 and Parkin in renal tissues compared with the control group, indicating PINK1/Parkin-mediated mitophagy activation. In contrast, treatment with SC and AMF markedly attenuated PINK1 and Parkin expression, suggesting mitophagy modulation. Figure 8B demonstrates that cisplatin treatment markedly increased LC3 and Beclin-1 protein expression while reducing p62 levels, reflecting altered autophagic activity. Conversely, SC and AMF treatments reversed these changes, suggesting regulation of autophagy in renal tissues. Figure 8C reveals marked cytochrome c and cleaved caspase-3 upregulation following cisplatin administration, indicating mitochondrial-mediated apoptotic signaling activation. These changes were significantly attenuated by SC and AMF treatments. Figure 8D shows marked Bax and caspase-3 upregulation, accompanied by reduced Bcl-2 expression in renal tissues following cisplatin treatment, indicating apoptosis induction. Treatment with SC and AMF markedly counteracted these changes by downregulating Bax and caspase-3 expression while upregulating Bcl-2 expression. Terminal deoxynucleotidyl transferase-mediated dUTP nick end labeling (TUNEL) staining further demonstrated a marked increase in TUNEL-positive cells following cisplatin treatment relative to untreated controls, reflecting enhanced apoptosis in renal tissues (Figure 8E). However, SC and AMF treatments significantly reduced the TUNEL-positive area, further confirming their protective effects against cisplatin-induced apoptosis.

## 3. Discussion

AKI is a major dose-limiting adverse effect of cisplatin chemotherapy that is associated with increased risk of chronic kidney disease and mortality [37]. The development of effective strategies to prevent or attenuate cisplatin-induced nephrotoxicity therefore remains an urgent clinical need. Oxidative stress and inflammation have been recognized as key mechanisms underlying cisplatin-induced AKI [38]. In this study, a mouse model of cisplatin-induced AKI was established to evaluate the protective effects of SC, with AMF as a reference treatment.

SC has been reported to possess various pharmacological activities, which may partly be attributed to its bioactive component adenosine, a nucleoside with anti-inflammatory, neuroprotective, anti-angiogenic, lipid-lowering, anticonvulsant, antioxidant, and immunomodulatory effects [39]. In our previous study, adenosine was identified and quantified as a representative marker compound of SC using HPLC-photodiode array detection (HPLC-PAD), with a retention time of 8.3 min and a relative content of 14.21 μg/mL [17]. In addition, accumulating evidence indicates that adenosine plays a critical role in AKI [40]. Adenosine signaling via its receptors (A1, A2A, A2B, and A3) regulates renal inflammation, hemodynamics, and cellular stress responses, while A1 and A2A receptor activation has been shown to attenuate renal injury by reducing inflammation, improving renal perfusion, and suppressing leukocyte infiltration [41]. Although pharmacokinetic analyses were not performed in the present study, previous investigations have demonstrated that adenosine and cordycepin can be detected in the bloodstream and various tissues following administration, indicating systemic exposure and in vivo metabolism. Furthermore, recent studies on *SC* polysaccharides suggest that orally administered polysaccharides are capable of being absorbed, although their absorption efficiency may be influenced by factors such as molecular weight, electrical charge, molecular conformation, and dosage [42]. Therefore, while the renal distribution of SC extract constituents was not directly evaluated in this study, the observed renoprotective effects following oral administration imply that the bioactive components of SC extract reached sufficient systemic concentrations to elicit biological activity in vivo. However, definitive evidence of renal bioavailability still requires future pharmacokinetic studies.

Cisplatin administration induced marked renal dysfunction, characterized by elevated serum CRE and BUN levels, increased kidney index, and body weight loss [39]. Histopathological analysis further revealed severe renal structural damage, including tubular epithelial injury, vacuolar degeneration, tubular dilation, necrosis, and inflammatory cell infiltration. In contrast, SC treatment markedly improved renal function and attenuated these histopathological alterations, indicating a protective effect against cisplatin-induced renal injury. A limitation of this study is that we did not directly evaluate whether SC extract affects cisplatin’s antitumor efficacy. Our previous data only show that SC alone is not cytotoxic to HepG2 and 293T cells; whether SC antagonizes cisplatin-induced cancer cell death remains unknown [17]. Future studies must include co-treatment experiments on relevant cancer cell lines to verify that the renoprotective effect does not come at the cost of reduced antitumor activity.

AKI is characterized by a strong inflammatory response, in which pro-inflammatory cytokines such as TNF-α, IL-1β, and IL-6 are critically involved in exacerbating renal damage [43]. In addition, as an important mediator of nitrosative stress, NO is also involved in the pathological process of renal injury and has been implicated in regulating programmed cell death [22]. The present findings suggest that attenuation of nitrosative stress and inflammatory responses is associated with SC’s renoprotective effects. To further explore the molecular mechanisms underlying these anti-inflammatory effects, the MAPK and TLR4/NF-κB signaling pathways were evaluated, as NF-κB signaling inhibition has been reported to attenuate cisplatin-induced renal injury [44]. Accumulating evidence indicates that excessive ROS production during cisplatin exposure leads to mitochondria-associated signaling and subsequent activation of MAPK pathways, including ERK1/2, JNK, and p38, which contribute to inflammatory responses and apoptosis [37]. The activation of these MAPK pathways has been closely associated with renal tubular injury and inflammatory progression in AKI. TLR4 is an important upstream mediator of inflammatory signaling in AKI. Upon activation, TLR4 cooperates with TLR2 to trigger MyD88-dependent signaling, leading to NF-κB and MAPK pathway activation and subsequent cytokine production [45]. The inhibition of MAPK and TLR4/NF-κB signaling contributes to the renoprotective effects of SC in cisplatin-induced AKI.

During AKI or CKD, neutrophils are rapidly recruited to inflammation sites. Once inside renal tissue, they produce large amounts of NO, which acts as a direct antibacterial agent and triggers the release of neutrophil extracellular traps (NETs) to capture and destroy invading pathogens [44]. Moreover, NO readily reacts with superoxide to form peroxynitrite (ONOO^−^), which exacerbates kidney tissue damage by inducing lipid peroxidation, protein nitration, and DNA damage. In addition, NO is a key regulator of neutrophil lifespan. Prolonged exposure to NO can induce neutrophil apoptosis by activating caspase cascades and reducing mitochondrial membrane potential. Following apoptosis, neutrophils are cleared by macrophages. If NO signaling fails to promote apoptosis, neutrophils may undergo secondary necrosis, releasing cytotoxic granules that further damage the renal parenchyma [45]. In CKD, systemic inflammation leads to persistent neutrophil activation, clinically manifested as an elevated neutrophil-to-lymphocyte ratio (NLR). A high NLR is an independent predictor of adverse renal outcomes, ESRD, and cardiovascular complications in CKD patients. Similarly, an increase in GSDMD-dependent NETs driven by the caspase pathway is strongly associated with renal fibrosis. The present findings support the involvement of the anti-inflammatory pathway in cisplatin-induced AKI and suggest that anti-inflammatory pathway enhancement is associated with SC’s renoprotective effects.

Oxidative stress is a key contributor to cisplatin-induced renal injury. Excessive ROS generation promotes lipid peroxidation, depletes endogenous antioxidant defenses, and disrupts redox homeostasis, as evidenced by increased TBARS levels and reduced GSH content. The Nrf2/HO-1 system is considered to be a major cellular defense mechanism against oxidative stress. Nrf2 plays an important role in cellular defense and in improving the removal of ROS by activating genes that encode phase II detoxifying and antioxidant enzymes, such as NAD(P)H-Quinone Oxidoreductase 1 (NQO1), glutamate–cysteine ligase catalytic subunit (GCLC), GPX, and glutathione S-transferases (GST) [38]. Under basal conditions, Nrf2 appears to be associated with actin-binding Keap1, which forms the Keap1-Nrf2 complex. The Keap1-Nrf2 complex prevents Nrf2 from entering the nucleolus, which promotes its proteasomal degradation. Upon treatment in cells with oxidants, including oxidative stress, changes occur due to the oxidation of thiol-sensitive amino acids that are present in the Keap1-Nrf2 complex and may drive Nrf2’s dissociation from Keap1. Nrf2 then translocates into the nucleus, where it binds to the antioxidant response element (ARE) of target genes, leading to enhanced antioxidant enzyme expression [46]. The present findings support the Nrf2/HO-1 pathway’s involvement in cisplatin-induced AKI and suggest that enhanced Nrf2-mediated antioxidant defenses are associated with SC’s renoprotective effects.

The CaMKK–AMPK–Sirt1 signaling axis plays a pivotal role in maintaining cellular energy homeostasis and regulating responses to oxidative stress [47]. CaMKK functions as an upstream kinase that activates AMPK in a Ca^2+^-dependent manner, while activated AMPK can regulate Sirt1 activity through modulating cellular NAD^+^ metabolism, thereby coordinating metabolic adaptation and stress resistance [30]. AMPK–Sirt1 pathway activation has been implicated in enhancing mitochondrial function and reducing oxidative damage in renal injury [48,49]. SC treatment increased CaMKK, p-AMPK, and Sirt1 expression, suggesting activation of the CaMKK–AMPK–Sirt1 signaling axis and its involvement in regulating energy metabolism and cellular stress responses during cisplatin-induced AKI. The PI3K/AKT pathway has been implicated in multiple pathological processes, including cellular stress adaptation, inflammatory regulation, and apoptosis [32].

The PI3K/AKT signaling pathway plays a pivotal protective role against AKI through multiple mechanisms. Primarily, this pathway is a key regulator of apoptosis. Activation of PI3K/AKT promotes cell survival and suppresses programmed cell death in renal tubular epithelial cells, a function mediated in part by inducing the nuclear export and deacetylation of FOXO3 (Forkhead Box O3). Furthermore, the pathway is crucial for modulating inflammation and oxidative stress. By activating downstream effectors like NRF2, PI3K/AKT signaling helps clear mitochondrial reactive oxygen species (ROS) and counteracts mitochondrial dysfunction and cell pyroptosis. Additionally, PI3K/AKT signaling can shift macrophage polarization toward an anti-inflammatory M2 phenotype, thereby attenuating the inflammatory response. The pathway also regulates autophagy and specialized cell death pathways like ferroptosis. Its downstream mTOR axis controls autophagic activity, which can either protect or exacerbate injury [34]. In certain contexts, activation of PI3K/AKT/mTOR inhibits autophagy to exert nephroprotective effects. Moreover, the pathway has been shown to inhibit ferroptosis, a form of iron-dependent cell death, thereby offering another layer of protection in AKI [35]. Finally, by reducing apoptosis, inflammation, and oxidative stress, the PI3K/AKT pathway also plays a role in mitigating the transition from AKI to CKD. In summary, the PI3K/AKT pathway acts as a central regulator of cell survival and homeostasis, making it a highly promising therapeutic target for AKI.

Apoptosis, autophagy, and mitophagy are closely involved in the progression of cisplatin-induced renal injury [1]. In toxin-induced AKI, apoptosis is predominantly mediated through the intrinsic mitochondrial pathway. Under cellular stress, mitochondrial outer membrane permeability (MOMP) increases, activating pro-apoptotic Bax and suppressing anti-apoptotic Bcl-2, thereby promoting cytochrome c liberation. Released cytochrome c interacts with apoptotic protease-activating factor-1 (Apaf-1) to form the apoptosome, which activates caspase-9 and downstream caspase-3, ultimately resulting in apoptotic cell death [50]. SC suppresses apoptosis by enhancing Bcl-2 expression while reducing Bax levels and downregulating caspase-3 protein levels, thereby exerting renoprotective effects in AKI models. Consistent with these findings, TUNEL staining demonstrated increased apoptotic DNA fragmentation in cisplatin-treated kidneys, whereas SC treatment significantly reduced the TUNEL-positive cells. These results provide morphological evidence supporting the anti-apoptotic effect of SC in cisplatin-induced AKI. Autophagy is involved in the cellular stress response. Under physiological conditions, autophagy plays a protective role by maintaining cellular homeostasis. However, in cisplatin-induced acute kidney injury, excessive or dysregulated autophagy may exacerbate tubular cell damage and crosstalk with apoptotic signaling, thereby contributing to renal cell death [51,52]. SC treatment modulated autophagy-related protein expression, suggesting attenuation of dysregulated autophagic processes in cisplatin-induced nephrotoxicity.

Mitophagy is a selective autophagic process that removes damaged mitochondria. The PINK1/Parkin pathway is recognized as a principal mechanism governing mitophagy in renal tubular cells and is closely involved in mitochondrial quality maintenance and cellular survival during renal injury [26,53]. PINK1 accumulates on the outer mitochondrial membrane upon mitochondrial depolarization and recruits Parkin from the cytosol, thereby activating its ubiquitin-ligase activity to promote dysfunctional mitochondria clearance [54]. Although mitophagy is generally considered a protective mechanism, excessive mitophagy may also be detrimental, leading to mitochondrial depletion, impaired ATP production, and increased cell death [27]. Notably, both SC and AMF reduced the cisplatin-induced elevation in PINK1/Parkin expression. While the renoprotective effects of AMF have been widely attributed to its antioxidant and -apoptotic properties, its influence on PINK1/Parkin-mediated mitophagy has not been extensively investigated.

The present findings suggest that mitochondrial quality control pathway modulation may also contribute to the protective effects of AMF in cisplatin-induced renal injury. There is, however, a lack of causal verification, underlining the need for future studies using inhibitors (e.g., PI3K/AKT, Nrf2, or PINK1/Parkin inhibitors) or gene knockdown techniques for confirmation.

## 4. Materials and Methods

### 4.1. Drugs and Reagents

All chemicals and reagents, including cisplatin, AMF, and other solvents, were purchased from Sigma-Aldrich (St. Louis, MO, USA). SerumBUN and CRE levels were determined using commercial assay kits supplied by HUMAN Diagnostics Worldwide (Wiesbaden, Germany). Mouse TNF-α, IL-1β, and IL-6 levels were measured using enzyme-linked immunosorbent assay (ELISA) kits (Max™ Set Deluxe, BioLegend, San Diego, CA, USA). For Western blotting, primary antibodies including B-cell lymphoma 2 (Bcl-2; GTX100064), phosphorylated-AKT (p-AKT; GTX128414), toll-like receptor 4 (TLR4; A5258), Kelch-like ECH-associated protein 1 (KEAP1; GTX60660), inhibitor of kappa B alpha (IκBα; GTX110521), NF-κB (GTX102090), sequestosome 1 (p62/SQSTM1; GTX100685), HO-1 (GTX101147), Sirt1 (GTX17532), phosphorylated-NF-κB (p-NF-κB; GTX50098), Bcl-2-associated X protein (Bax; GTX109683), glutathione peroxidase 3 (GPx3; AV41491), AKT (GTX121937), cytochrome c (GTX108585), and catalase (GTX110704) were acquired from GeneTex (San Antonio, TX, USA). Antibodies against phosphorylated-c-Jun N-terminal kinase (p-JNK; 9255s), Beclin-1 (3738s), microtubule-associated protein 1 light chain 3 beta (LC3B; 12741s), AMP-activated protein kinase (AMPK; 5831s), phosphorylated-AMPK (p-AMPK; 2535s), phosphorylated-calcium/calmodulin-dependent protein kinase kinase 2 (p-CAMKK2; 12818s), phosphorylated-IκBα (p-IκBα; 2859s), phosphorylated-p38 (p-p38; 4631s), and extracellular signal-regulated kinase (ERK; 4695S) were purchased from Cell Signaling Technology (Beverly, MA, USA). Antibodies against JNK (06-748) and PI3K (06-195) were purchased from Merck Millipore (Burlington, MA, USA). Antibodies, including phosphorylated-ERK (p-ERK; 44-680G), were purchased from Invitrogen (Carlsbad, CA, USA). Antibodies against PTEN-induced kinase 1 (PINK1, A7131), Parkin (A0968), cysteine-aspartic acid protease-3 (caspase-3; A16793), and Nrf2 (A1244) were purchased from Abclonal (Wuhan, China). Antibodies against p38 (ab31828) were purchased from Abcam (Cambridge, UK). Antibodies against cleaved caspase-3 (ARG66671) were purchased from Arigo Biolaboratories (Hsinchu, Taiwan). Antibodies against phosphorylated-PI3K (p-PI3K; E-AB-20966) and CAMKK2 (E-AB-30731) were purchased from Elabscience (Wuhan, China). Antibodies against superoxide dismutase 1 (SOD1; 3458-100) were purchased from BioVision (Milpitas, CA, USA). Primary antibodies were generally used at a dilution of 1:1000, except for HO-1 (1:500) and p-JNK (1:2000). β-actin (1:1000) was used as an endogenous control protein. The protein assay kits used in this study were supplied by Bio-Rad Laboratories Ltd. (Watford, UK).

### 4.2. Fungus Material

SC extract was provided by Tsairder Biotechnology Co., Ltd. (Taichung, Taiwan). Dried SC (100 g) was pulverized and passed through an 80-mesh sieve before extraction with distilled water at 65 °C for 1 h. The resulting extract was filtered and concentrated under reduced pressure at 40 ± 5 °C using a rotary evaporator, then freeze-dried to yield a powder, which was stored at −20 °C until further use.

### 4.3. Animal Treatment

Male ICR mice (6–8 weeks old, weighing 20–25 g) were obtained from BioLASCO Taiwan Co., Ltd (Taipei, Taiwan). Before the experiment, mice were acclimated for 1 week under controlled environmental conditions, including a 12 h/12 h light/dark cycle, a temperature of 23 ± 2 °C, and a relative humidity of 50–60%. All animal procedures and care were carried out in accordance with the guidelines of the Animal Management Committee of China Medical University (IACUC approval no.: CMUIACUC-2025-286).

The mice were randomly divided into five groups (*n* = 6 per group): control, cisplatin (20 mg/kg body weight, i.p.), amifostine (AMF; 200 mg/kg, i.p.) + cisplatin, SC (0.5 g/kg, i.g.) + cisplatin, and SC (1 g/kg, i.g.) + cisplatin. All mice were acclimated to the experimental environment for one week before treatment. SC was administered by oral gavage (i.g.) once daily for 10 consecutive days. Control mice were orally administered an equivalent volume of saline. On day 7, AKI was induced by a single intraperitoneal injection of cisplatin (20 mg/kg body weight) in the cisplatin and SC-treated groups, administered 1 h after SC treatment. On day 10, mice were sacrificed, and whole blood samples were collected. Blood samples were centrifuged at 4 °C (2000× *g* for 15 min) to obtain serum, which was stored at −20 °C until further analysis. Kidneys were immediately harvested for subsequent experiments. During the study period, clinical symptoms were assessed twice daily. Body weight was recorded daily, and the average for each group was calculated.

### 4.4. Serum Creatinine and Urea Nitrogen Measurements

CRE and BUN levels were measured using a chemical analyzer (Cobas Mira Plus; Roche Diagnostics, Germany) according to the manufacturer’s instructions.

### 4.5. Histopathological Analysis

Kidney samples were fixed in 10% formalin, embedded in paraffin wax, and sectioned into 5 μm thick slices. The sections were stained with hematoxylin and eosin (H&E) and examined under a light microscope (Nikon Eclipse TS100, Tokyo, Japan), with representative images captured. The severity of renal injury was determined by assessing epithelial damage in the renal cortical tubules [55].

### 4.6. Kidney/Body Mass Index Assessment

Body weight was recorded before euthanasia. Following sacrifice, the kidney tissues were carefully isolated and weighed. The kidney index was calculated as follows: (kidney weight/body weight) × 100%.

### 4.7. Nitrite Assay

Nitrite levels were quantified using the Griess colorimetric assay as previously described [56]. In brief, 100 μL of serum was transferred into a 96-well plate and mixed with an equal volume of Griess reagent. After incubation at room temperature for 10 min, absorbance was detected at 540 nm using a microplate reader (Molecular Devices, Orleans Drive, Sunnyvale, CA, USA). Nitrite levels were used as an indicator of NO production.

### 4.8. Cytokine Assay

Commercial ELISA kits (BioLegend, San Diego, CA, USA) were used to quantify serum TNF-α, IL-6, and IL-1β levels according to the supplier’s instructions. Absorbance at 450 nm was detected using a microplate reader, and each sample was assayed in duplicate. Cytokine concentrations were calculated based on standard curves generated using the corresponding reference standards.

### 4.9. Lipid Peroxidation Assays

Kidney tissues were homogenized in an appropriate lysis buffer and centrifuged, and the resulting supernatant was collected for biochemical analysis. MDA, an indicator of lipid peroxidation, was measured using a (TBARS assay [57]. Kidney extracts were mixed with TBA reagent and incubated at 90 °C for 45 min, and then subjected to n-butanol extraction and centrifugation as previously described [58]. Supernatant absorbance was measured at 535 nm, and the results were expressed as MDA equivalents (nmol/mg protein).

### 4.10. Glutathione Estimation

GSH levels were measured using the DTNB (5,5′-dithiobis (2-nitrobenzoic acid)) assay. Tissue homogenates were treated with 10% trichloroacetic acid, and centrifuged at 1500× *g* and 4 °C for 10 min, and the supernatant was reacted with DTNB. GSH concentrations were derived from a standard curve following absorbance measurement at 412 nm [59].

### 4.11. Western Blot Analysis

For Western blot analysis, kidney tissues were homogenized and lysed in RIPA buffer containing protease inhibitors, and the lysates were then centrifuged at 10,000× *g* for 10 min. Protein concentrations were analyzed with a Bio-Rad protein assay kit (Bio-Rad, Hercules, CA, USA). Equivalent amounts of protein were resolved by 12% SDS–polyacrylamide gel electrophoresis (SDS-PAGE), followed by membrane transfer. Following the blocking step, membranes were probed with primary antibodies (1:1000) at 4 °C overnight, and then incubated with appropriate horseradish peroxidase (HRP)-conjugated secondary antibodies (1:5000) (Sigma, St. Louis, MO, USA). Protein bands were detected using an enhanced chemiluminescence (ECL) detection system (Amersham International plc., Buckinghamshire, UK), and images were captured with KODAK Molecular Imaging Software (Eastman Kodak Company, Rochester, NY, USA).

### 4.12. TUNEL Staining

Kidney tissues were fixed in 10% neutral buffered formalin, paraffin-embedded, and sliced into 5 μm thick sections. Apoptotic cells in kidney sections were detected using TUNEL staining. TUNEL staining was carried out using a commercial apoptosis detection kit (Roche Molecular Biochemicals, Indianapolis, IN, USA) according to the manufacturer’s instructions. Histological sections were visualized and photographed using a light microscope (Nikon Eclipse TS100, Tokyo, Japan).

### 4.13. Statistical Analysis

Results were statistically analyzed, with values presented as mean ± standard deviation (S.D.). Student’s *t*-test was employed for pairwise comparisons, and one-way ANOVA followed by Tukey’s post hoc analysis was used to evaluate differences among multiple treatments. A *p*-value < 0.05 denoted statistical significance.

## 5. Conclusions

In conclusion, SC ameliorated cisplatin-induced AKI, as evidenced by the improved renal function and reduced histopathological damage, as well as attenuated oxidative stress and inflammatory responses. Mechanistically, SC modulated multiple signaling pathways—including MAPK, TLR4/NF-κB, PI3K/AKT, Nrf2/HO-1, and the CaMKK–AMPK–Sirt1 axis—and regulated apoptosis, autophagy, and PINK1/Parkin-mediated mitophagy, suggesting improved mitochondrial homeostasis. These results support SC’s renoprotective role via the integrated regulation of oxidative stress, inflammation, and mitochondrial quality control. Furthermore, in order to define their coordinated roles in AKI progression, the interplay among apoptosis, autophagy, and PINK1/Parkin-mediated mitophagy warrants further investigation. Taken together, SC represents a functional material with potential value in mitigating cisplatin-induced nephrotoxicity.

## Figures and Tables

**Table 1:** Kidney weight and index among experimental groups. Data are presented as mean ± SD (*n* = 6).

Group	Body Weight (g)		Kidney Weight (g)	Kidney Index (mg/g)
	Initial	Final		
Control	34.40 ± 0.75	38.73 ± 1.11	0.47 ± 0.03	1.21 ± 0.04
Cisplatin (20 mg/kg)	34.60 ± 1.05	30.16 ± 1.00 ^###^	0.68 ± 0.05 ^###^	2.24 ± 0.10 ^###^
Cisplatin (20 mg/kg) + Amifostin (200 mg/kg)	34.60 ± 0.62	32.87 ± 1.13 **	0.54 ± 0.05 **	1.65 ± 0.10 ***
Cisplatin (20 mg/kg) + SC (0.5 g/kg)	34.70 ± 0.64	32.33 ± 1.18 **	0.57 ± 0.08 *	1.75 ± 0.18 ***
Cisplatin (20 mg/kg) + SC (1 g/kg)	34.28 ± 1.26	34.46 ± 1.73 **	0.49 ± 0.05 ***	1.42 ± 0.08 ***

^###^ *p* < 0.001 vs. control group; * *p* < 0.05, ** *p* < 0.01 and *** *p* < 0.001 vs. cisplatin group.

## Data Availability

The original contributions presented in this study are included in the article. Further inquiries can be directed to the corresponding author.
